# Characterisation of Nanocellulose Types Using Complementary Techniques and Its Application to Detecting Bacterial Nanocellulose in Food Products

**DOI:** 10.3390/nano15201565

**Published:** 2025-10-14

**Authors:** Otmar Geiss, Ivana Bianchi, Ivana Blazevic, Guillaume Bucher, Hind El-Hadri, Francesco Fumagalli, Jessica Ponti, Chiara Verra, Josefa Barrero-Moreno

**Affiliations:** European Commission, Joint Research Centre (JRC), 21027 Ispra, Italyjosefa.barrero@ec.europa.eu (J.B.-M.)

**Keywords:** nanocellulose, crystalline nanocellulose, bacterial nanocellulose, fibrillated nanocellulose, SCOBY, kombucha, nata de coco, AF4-MALS, pyrolysis GC-MS

## Abstract

Nanocellulose has attracted significant attention in recent years due to its distinctive properties and vast potential applications across various fields. This study encompasses two distinct yet interconnected activities: the characterisation of eight different types of nanocellulose test materials, including crystalline, fibrillated, and bacterial nanocellulose, using a range of analytical techniques such as dynamic light scattering (DLS), asymmetric flow field-flow fractionation (AF4) coupled to multi-angle light scattering (MALS) and DLS, and transmission electron microscopy (TEM), and a focused case study employing a tiered analytical approach to identify bacterial nanocellulose in commercially available food products like pudding and drinks with nata de coco, SCOBY, and kombucha. The results demonstrate that different types of nanocellulose can be distinguished by their unique physicochemical properties using a combination of analytical techniques. This finding was used for the identification of bacterial nanocellulose in food products by combining pyGC-MS for cellulose identification, TEM for nanosize range determination, and XRD for crystallinity analysis to distinguish between bacterial and fibrillated nanocellulose. The study advances fundamental understanding of nanocellulose and provides tools to facilitate potential future regulatory compliance.

## 1. Introduction

Green, renewable, and sustainable materials are becoming increasingly important as the world shifts towards a more environmentally conscious and responsible future. The depletion of non-renewable resources, coupled with growing consumer demand for eco-friendly products, has led to a surge in the development and adoption of sustainable materials. Furthermore, advances in technology have made it possible to create new materials with improved performance, durability, and cost-effectiveness, while also reducing waste and the environmental impacts associated with traditional materials. In this context, cellulose has been revealed to be a promising candidate with regard to its abundant availability from various resources [1]. Cellulose can be functionalised in many ways due to its chemical structure, which features highly reactive hydroxyl groups that can participate in various chemical reactions, allowing for the introduction of a wide range of functional groups. The versatility of cellulose is further enhanced by its ability to undergo reactions under different conditions, making it a highly adaptable and biocompatible material for various applications, from biomedical, such as wound dressings, to environmental fields like water treatment [2].

On a much smaller scale, cellulose can be processed into a material with distinct characteristics, known as nanocellulose. Nanocellulose has been gaining attention in recent years due to its unique properties and potential applications in various fields. Nanocellulose can be categorised into nanostructured materials and nanofibers. The second category comprises cellulose nanofibrils, cellulose nanocrystals, and bacterial nanocellulose [3]. Cellulose nanocrystals exhibit high crystallinity and a rod-like, cylindrical shape and are formed via acidic hydrolysis; cellulose nanofibrils are formed through mechanical processing and consist of long, entangled fibres with a high aspect ratio, moderate crystallinity, and high viscosity in suspension due to strong hydrogen bonding; and bacterial nanocellulose features ribbon-shaped nanofibers organized in a three-dimensional, entangled network, generated by bacterial fermentation. Crystalline nanocellulose, known for its structural rigidity and thermal stability, is widely used in pharmaceuticals as a tablet excipient and in composites for reinforcement [1]. Fibrillated nanocellulose, derived from mechanical or chemical breakdown of fibres, finds applications in high-absorbency paper products, biomedical wound dressings, and as a stabilizer in foods and coatings. Bacterial nanocellulose, produced by microbial fermentation, is prized for its high purity, nanoscale porosity, and mechanical strength, making it ideal for advanced biomedical applications like tissue engineering scaffolds, flexible electronics, and biodegradable packaging materials [4]. In the food sector, due to the above-described characteristics, nanocellulose presents significant potential for use as food additives (stabiliser, emulsifier, thickener, bulking agent, and glazing agent) [5,6]. Nanocellulose can also be considered as an engineered nanomaterial, a category of novel food defined in Regulation (EU) 2015/2283 [7] as “any intentionally produced material that has one or more dimensions of the order of 100 nm or less or that is composed of discrete functional parts, either internally or at the surface, many of which have one or more dimensions of the order of 100 nm or less, including structures, agglomerates or aggregates, which may have a size above the order of 100 nm but retain properties that are characteristic of the nanoscale”. However, nanocellulose and its modified forms are currently not authorised for use as novel foods or food additives in the European Union (EU). Since it is currently not authorised in the EU, substances such as nanocellulose consisting of engineered nanomaterials as defined in point (f) of Regulation (EU) 2015/2283 require pre-market authorisation as a novel food. Alternatively, a food additive authorisation may be required if nanocellulose is added to food for a technological purpose (Regulation (EC) No 1333/2008) [8]. For both pathways, comprehensive data on the material’s physical and chemical characteristics are essential to conduct a robust risk assessment and ensure safe use.

This study encompasses two distinct yet interconnected activities: (1) the characterisation of eight different types of nanocellulose test materials, including crystalline, fibrillated, and bacterial nanocellulose, using a range of analytical techniques such as dynamic light scattering, asymmetric flow field-flow fractionation, and transmission electron microscopy, which adds to the already existing knowledge in the field and may be useful for future risk evaluation studies, and (2) a focused case study employing a tiered analytical approach combining pyGC-MS, TEM, and X-ray diffraction to identify and characterise bacterial nanocellulose in commercially available food products. While the former establishes baseline material properties, the latter applies these insights to a practical context and could enhance future compliance testing protocols for bacterial nanocellulose contained in novel food products within the European Union. The study thus advances both foundational knowledge of nanocellulose and practical tools to support potential future regulatory compliance.

## 2. Materials and Methods

This study has two main components. In the first, we conducted a systematic characterisation of eight types of aqueous nanocellulose test material suspensions, which were used in their native state without undergoing further purification or extraction. Second, we focused on a case study of three types of bacterial nanocellulose found in real food products: nata de coco (in pudding and the Mogu Mogu drink) and SCOBY/kombucha. These required extraction and purification before analysis. Since some sample preparation methods and analytical techniques differed, they are reported separately.

### 2.1. Nanocellulose Test Materials

#### 2.1.1. Description of Nanocellulose Test Materials

This study investigated eight different types of aqueous nanocellulose suspensions deemed relevant for food-related applications and associated human exposure in a recent EFSA-funded project [9]. The eight test materials consisted of three nanocrystal types, two bacterial nanocellulose types, and three nanofiber types, as outlined in Table 1. The origin of biological materials and the methods used to prepare nanocellulose significantly influence its physicochemical properties, such as particle size, shape, aspect ratio, surface charge, chemical composition, crystallinity, and polydispersity. These characteristics, in turn, directly affect the environmental behaviour and potential risks associated with nanocellulose, underscoring the importance of investigating a diverse range of materials.

For example, TEMPO-oxidized nanocellulose is distinguished by its anionic surface charge (derived from carboxylate groups) and colloidal stability, achieved through chemical oxidation and mild processing. This method preserves long, thin fibrils with high crystallinity. By contrast, cellulose nanocrystals (CNCs) are short, highly crystalline particles produced via acid hydrolysis and often functionalized with sulfonic acid groups. Cellulose nanofibrils (NFCs), on the other hand, are long, flexible fibrils generated through mechanical processing. While they provide structural reinforcement, their colloidal stability typically requires the addition of surfactants.

Bacterial nanocellulose (BNC) forms a three-dimensional porous network composed of ultrapure, highly crystalline cellulose. Valued for its exceptional mechanical strength and water-holding capacity, BNC stands out as it is synthesized biologically, bypassing the need for harsh chemicals or mechanical energy used in chemically or mechanically derived nanocelluloses.

The NFC and CNC materials were obtained from commercial providers (including academic-associated centres), while the bacterial nanocellulose materials were donated by researchers involved in industry-related R&D activities.

#### 2.1.2. Sample Preparation of Nanocellulose Test Materials

Prior to analysis, the raw sample suspensions were diluted, sonicated, and filtered to achieve stable and de-agglomerated suspensions. The optimisation protocol was largely adapted from the EFSA Project on the use of New Approach Methodologies (NAMs) for the hazard assessment of nanofibers [9]. In brief, the raw materials with IDs 1, 2, and 6 listed in Table 1 were diluted in ultrapure water to achieve a final concentration of nanocellulose of 2% (*w*/*w*). Raw suspensions with IDs 3, 4, 5, 7, and 8 (bacterial and nanofibrillated nanocellulose types) were diluted to reach concentrations of 0.3% (*w*/*w*). This became necessary because at higher concentrations, the viscosity was so high that it could not be transferred. These diluted suspensions were then transferred into 15 mL Falcon^TM^ tubes and sonicated with a probe-sonicator (VibraCell VCX-130, 3 mm tip, Sonics & Materials Inc, Newton, CT, USA). The sonication conditions were optimised on one material of each nanocellulose type (CNC1, NFC2, and BNC1) by applying various amounts of (delivered) sonication energy (7 kJ, 10 kJ, and 12 kJ). For all tested nanocellulose types, 7 kJ was found to be sufficient for an optimal dispersion (see Section 3.1.1). Therefore, all other materials were sonicated, delivering an energy of 7 kJ in approximately 9 min of sonication, setting the amplitude at 90%. The delivered acoustic power was determined following the approach described by Taurozzi et al. [10] and was found to be 12.5 W (SM1). Samples were immersed in an ice bath during the sonication process to prevent overheating. These sonicated intermediate suspensions were filtered through a 0.45 µm Polyvinylidene difluoride (PVDF) 13 mm syringe filter (Millex-HV filter unit, P/N SLHV013L, Sigma Aldrich, St. Louis, MO, USA). Additional sample preparation steps varied depending on the analytical technique used and are described in the following sections.

##### Determination of Sample Recovery During Filtration of Nanocellulose Test Materials

The sample recovery after filtration was determined gravimetrically using an ultra-microbalance (Model XR2TU, Mettler Toledo, Greifensee, Switzerland) for materials CNC1, NFC1, NFC2, and BNC1. To this end, 50 µL of the unfiltered and filtered suspensions described in Section 2.1.2 were transferred into small metal cups (80 µL Eco-Cup LF, P/N PY1-EC80F, Frontier Lab, Koriyama, Japan), which had previously been weighed (tare). The water was then evaporated overnight (15 h) in a ventilated oven set at 40 °C. The amount of nanocellulose in the cup was obtained by differential weighing (cup after drying the suspension—tare of the empty cup). The recovery was calculated by measuring the amount of cellulose before and after filtration. Three replicates were determined for each sample material.

#### 2.1.3. Dynamic Light Scattering (DLS, Batch Mode) on Nanocellulose Test Materials

Samples were analysed with a Malvern Zetasizer Nano-S (Malvern Panalytical, Malvern, UK). Prior to analysis, the samples were further diluted with a 5 mM NaCl solution to achieve a final nanocellulose concentration of 0.05% (*w*/*w*). The diluted suspensions were then transferred to disposable cuvettes. The settings outlined in Table 2 were adjusted in the instrument’s software. Three replicate measurements were run for each sample. Samples were analysed before and after the filtration step.

#### 2.1.4. Asymmetric Flow Field Flow Fractionation (AF4) Coupled to MALS and DLS on Nanocellulose Test Materials

The three types of crystalline nanocellulose (CNC1, CNC2, and CNC3) and the TEMPO-oxidised cellulose nanofibrils (NFC1) were analysed by coupling the asymmetric flow field flow fractionation (AF4) system with a multi-angle light scattering detector (MALS), a DLS in flow mode, a UV detector, and a refractive index (RI) detector. The sonicated samples (Section 2.1.2) were diluted with a 1 mM NaCl solution to reach a final concentration of 0.3% (3000 ppm).

The AF4 system employed was comprised of an Eclipse Dualtec separation system (Wyatt Technology Europe GmbH, Dernbach, Germany) and an Agilent 1260 Infinity high-performance liquid chromatograph (Agilent Technologies, Santa Clara, CA, USA) equipped with a degasser (G1322A), an isocratic pump (G1310B), autosampler (G1329B), and a multiple wavelength (MWD) detector (G1365C) set at 230 nm. In the Eclipse SC separation channel, regenerated cellulose membranes (10 kDa) and a spacer height of 350 μm were used. The eluent was a 1 mM sodium chloride solution. The detector flow rate was set at 0.5 mL min^−1^ with an injection volume of 50 µL, corresponding to 150 µg of cellulose. The separation settings were based on a study conducted by Mukherjee and Hackley [11]: (a) Elution 0–2 min; (b) Focus: 2–4 min (Focus flow: 2.0 mL min^−1^); (c) Focus + Inject: 4–7 min (Focus flow: 2.0 mL min^−1^); (d) Focus: 7–10 min (Focus flow: 2.0 mL min^−1^); (e) Elution 10–70 min (Cross-flow from 0.8 mL min^−1^); (f) Elution 70–75 min (Cross-flow 0.0 mL min^−1^). The outlet of the MWD detector was connected to a DAWN 8+ HELEOS II multi-angle light scattering detector (MALS) operating with a 658 nm laser and an Optilab RI-detector (Wyatt Technology Europe). A DLS (Zetasizer Nano-S, Malvern Panalytical, Malvern, UK) with an installed quartz flow-cell (ZEN0023) was also used in this study in flow mode. Sodium chloride (1 mM) was set as the dispersant, the temperature was set to 25 °C, while the attenuation was set to 11. ‘General purpose (normal distribution)’ was chosen as the analysis model. MALS data were analysed using ASTRA software (version 6.1.7.17). The hydrodynamic radius was determined by dynamic light scattering (DLS), whereas the radius of gyration was derived from MALS data applying the Zimm model. Additionally, the rod length was calculated by applying the rod model. Each sample was injected in duplicate (*n* = 2).

Recovery of the injected material is one of the performance criteria outlined in ISO/TS 21362 [12] and should be higher than 70%. The recovery was determined by comparing the signal area obtained by the RI-detector of the sample following the separation described above and the signal area obtained by direct injection (no cross-flow). The recovery was determined on three replicates for each sample material.

#### 2.1.5. Transmission Electron Microscopy (TEM) on Nanocellulose Test Materials

The already diluted solutions, sonicated with an energy of 7 kJ as described in Section 2.1.2, were further diluted with ultrapure water at a ratio of 1:1000 for crystalline and TEMPO-oxidized samples, and 1:2000 for bacterial and fibrillated samples. Subsequently, 3 µL aliquots were manually spotted on Formvar Carbon-coated 200 mesh copper grids (Agar Scientific, Stansted, UK) and left to dry overnight. The next day, the grids were treated with 5% uranyl acetate for positive staining. The dried grids were imaged with a JEOL JEM-2100 HR-transmission electron microscope at 120 kV (JEOL, Basiglio, Italy) coupled to an X-Flash EDX Detector 5030 (Bruker, Milan, Italy). Using ImageJ software (ImageJ Fiji, Version 5, National Institutes of Health, Bethesda, MD, USA), the size distributions of 100 particles were manually measured and analysed. The choice of 100 particles was based on the sample’s relatively low size distribution variability and the practical limitations of manual measurement. This number was considered adequate to estimate central tendencies and characterise the overall size distribution.

### 2.2. Food (Related) Products Containing Bacterial Nanocellulose

For this study, four products suspected to contain bacterial (nano)cellulose were purchased. Two of these products included nata de coco among the ingredients, the third was a pad of ‘Symbiotic Culture of Bacteria and Yeast’ (SCOBY), the fermentation starter commonly employed in kombucha beverage preparation. The fourth product was a commercially available kombucha tea that had already undergone fermentation. Nata de coco is a popular foodstuff in certain Asian countries, produced through the fermentation of coconut water. Following an extended period of fermentation by acetic acid bacteria, it undergoes a transformation into a jelly-like cellulose-based hydrogel, characterised by a soft, chewy, and gelatinous texture. Consequently, nata de coco can be regarded as a type of bacterial (nano)cellulose [13]. Despite its popularity, relatively few products containing nata de coco are currently available on the European market. Two such products were sourced for this study: the first, a gelee pudding containing nata de coco cubes, was purchased online through Amazon (Cocon Gelee pudding nata de coco aux fruits exotiques, distributed by MAI DISTRIBUTION-Partenaire de THANH BINH JEUNE, Paris, France). The second product, Mogu Mogu, a fruit juice beverage with chewy nata de coco jelly cubes, advertised to add a distinctive texture to the drink, was obtained from a large supermarket in Italy (Carrefour market, Rome, Italy). The third product used in this study was a SCOBY starter culture purchased online through Amazon (KombuChic, Kombucha Scoby Starter Kit, Elisabeth Weber, Saint-Francois-Lacroix, France). The SCOBY is a biofilm that is home to a variety of microorganisms, including bacteria and yeast, which work together to ferment the tea and create the characteristic tangy flavour and fizzy texture of the kombucha drink. It is typically added to a sweetened black or green tea solution, where it consumes the sugars and catalyses the production of the fermented drink [14,15]. The fourth product, kombucha green tea, was selected for investigation to determine whether it retained residues of bacterial nanocellulose fibres after undergoing the fermentation process. This product was purchased from a ‘Natura Sì’ store located in Varese, Italy. Images of the four products are provided in the Supplementary Materials (SM2). The declared ingredients of the three tested products are listed in Table 3.

#### 2.2.1. Extraction and Clean-Up Procedures

(a) Extraction and clean-up of nata de coco from pudding

The pudding contained nata de coco cubes of an approximate size, ranging from 0.5 to 1 cm^3^. These cubes were isolated from the remaining product and washed with ultrapure water to remove any residual pudding from their surface. Three to five cubes were subsequently transferred into a 50 mL Falcon^TM^ tube and suspended in 30 mL of ultrapure water. This suspension was then ground for 5–10 s using an Ultra Turrax disperser (IKA 518 digital Ultra Turrax with S18N-19G dispersion tool, IKA-Werke GmbH & Co. KG, Staufen, Germany). The grinding time was limited to a few seconds to minimize potential effects on texture. The homogenised suspension was bath-sonicated for 15 min at 25 °C and then centrifuged at 3428 rcf (Eppendorf Centrifuge 5920R) to separate the solid and liquid phases. The upper (sugar-containing liquid) phase was then removed, and 30 mL of fresh ultrapure water was added. The tube was then vortexed until a homogeneous suspension was obtained. This washing procedure was repeated four times. After the fifth washing step, the jelly-like precipitate was transferred into 5 mL Eppendorf tubes and freeze-dried. For comparison purposes, one cube was freeze-dried without previously applying the above-described washing procedure.

(b) Extraction and clean-up of nata de coco from Mogu Mogu drink

Differences in the behaviour of nata de coco cubes during the clean-up procedure were observed between product A (Mogu Mogu drink) and product B (pudding). Following homogenisation and suspension in ultrapure water, a gel-like phase formed in product A, hindering the separation of the water phase from the nata de coco phase during centrifugation. As an alternative clean-up procedure, the isolated entire cubes (around 5) were transferred to a 300 mL beaker and subjected to mechanical stirring for 2 days, with the water being changed 3–4 times daily. Over this period, the initially dark-red cubes underwent a significant loss of colour, indicating successful clean-up. Subsequently, the washed cubes were freeze-dried prior to characterisation using pyGC-MS, TEM, and XRD.

(c) Extraction and clean-up of SCOBY starter culture

The entire SCOBY jelly-like pad was transferred to a 300 mL beaker and subjected to a washing procedure in ultrapure water, which involved changing the water 2–4 times daily over a period of 2 days. During this time, the initially brown pad underwent a significant loss of colour, resulting in a predominantly white appearance. Following completion of the washing procedure, the pad was cut into smaller pieces, transferred to 5 mL vials, and subsequently freeze-dried prior to characterisation using pyGC-MS, TEM, and XRD.

(d) Extraction and clean-up of sediment in Kombucha tea

At the bottom of the kombucha tea bottle, a small quantity of solid residue was visible. This sediment was concentrated by repeatedly adding 50 mL aliquots from the 100 mL kombucha tea bottle to a 50 mL Falcon^TM^ centrifuge tube, centrifuging the solid content to the bottom of the tube, and discarding the aqueous supernatant. To facilitate the subsequent steps, the accumulated solid sediment was then resuspended in ultrapure water and transferred to a smaller 15 mL Falcon^TM^ tube. In the next step, water-soluble sugars were removed by repeatedly (10 times) separating the solid component of the suspension from the aqueous (sugar-containing) supernatant through centrifugation and resuspension in fresh ultrapure water, followed by vortexing until a homogeneous suspension was achieved. The centrifugation conditions are detailed in Section 2.2.1.a. After this washing step, the dark, wet solid was transferred to a 5 mL Eppendorf^TM^ tube and left to dry overnight at 40 °C. The centrifugation conditions are described in Section 2.2.1.a.

#### 2.2.2. Pyrolysis GC-MS on Food Samples

Approximately 150 µg of the freeze-dried materials (for product B, both washed and not-washed) and approximately 200 µg of the oven-dried kombucha tea sediment were transferred into 80 µL pyrolysis cups (Eco-Cup LF, P/N PY1-EC80F, Frontier Laboratories, Funabashi, Japan). Pyrolysis GC-MS was performed using a Frontier Multi-Shot EGA/PY3030D microfurnace pyrolysis unit (Frontier Laboratories, Japan) coupled with an AS2020E auto-sampler operated in ‘single shot’ mode. This pyrolyser was coupled to an Agilent 8890/5977B GC-MS system (Agilent Technologies, Santa Clara, CA, USA). The pyrolyser furnace temperature was set to 600 °C, with the interface at 280 °C. For the GC-MS system, a split–splitless injector was used at a split ratio of 1:50, with the injector temperature set at 280 °C. Helium served as the carrier gas, with a constant flow rate of 1 mL min^−1^. Compounds were separated using an Agilent HP-MS Ultra inert-capillary column (Column length 30 m, width 0.25 mm, film 1 µm, product code 19091S-233UI; Agilent Technologies, Santa Clara, CA, USA). The oven program followed this sequence: 40 °C (hold for 5 min); ramped up by 20 °C min^−1^ until 315 °C was reached, with a subsequent 10 min hold. The total runtime was 28.75 min. The temperature of the MS ion source was set at 230 °C, and the quadrupole temperature at 150 °C. Samples were analysed in total ion mode (TIC). Mass spectra were analysed with Agilent Mass Hunter and the NIST MS Search library 2.4 installed thereon. Samples were analysed in duplicate.

#### 2.2.3. Transmission Electron Microscopy (TEM) on Food Samples

Approximately 10 mg of the (freeze-)dried material was transferred to a 1.5 mL Eppendorf tube, where it was suspended in ultrapure water and ultrasonicated using a Vial Tweeter (Hielscher, model UIS250v, Teltow, Germany) for 15 min at 75% amplitude and 50% cycle time. However, despite the sonication, the material failed to disperse homogeneously due to its inherent tissue-like structure. Consequently, an aliquot was taken from the edge of the floating material for spotting onto the copper grid, and the subsequent analysis was carried out as described in Section 2.1.5.

#### 2.2.4. X-Ray Diffraction (XRD) on Food Samples

X-ray diffraction (XRD) was employed to characterise the structure and crystallinity of three out of the four food samples. Three separate replicates of each material were prepared from the stock and measured independently. The dry sample materials were transferred into specimen holder rings (Product code C79298A3244D81, Bruker, Billerica, MA, USA) and pressed to form a homogeneous layer. This was done for all materials, with the exception of the dried kombucha tea residue, for which the requisite amount for analysis could not be obtained. XRD patterns were recorded using a D8 Advance diffractometer (Bruker, Billerica, MA, USA) in a parallel beam configuration. X-ray copper source settings were: I = 40 mA and V = 40 kV, wavelength Cu Kα 1.54 Å. The source was used in line focus. Incident optics inserted along the primary beam path and used for all measurements were, in order, a Göbel mirror, 2.5° Soller slits set, and a 6 mm axial slit. The sample was kept fixed during measurement. Receiving optics inserted along the diffracted beam path were a 2.5° Soller slits set and a 6 mm receiving slit. An acquisition scan was performed in 2θ/θ, symmetric (coupled) mode. Measured 2θ range spanned from 10° to 60°, the step size was 0.01°, and the analysis time per step was 2 s. Crystallinity and crystallite sizes were calculated using methods described in Artusio et al. [16]. Diffractometer calibration, alignment, and measurement procedures were performed according to guidelines from [17].

## 3. Results and Discussion

As mentioned earlier, this study encompasses two distinct but related activities: the characterisation of eight different types of nanocellulose test materials and a focused case study using a multi-step analytical approach combining pyGC-MS, TEM, and X-ray diffraction to identify and characterize bacterial nanocellulose in commercially available food products. The results are reported separately to highlight their distinct objectives—the former serves to define material properties and the latter to address application-specific challenges in the food sector.

### 3.1. Nanocellulose Test Materials

#### 3.1.1. Optimisation of the Sonication Conditions During the Sample Preparation

As outlined in Section 2.1.2, unfiltered suspensions of CNC1, NFC2, and BNC2 were subjected to different levels of sonication energy (no sonication = 0 kJ, 7 kJ, 10 kJ, and 12 kJ). Dynamic light scattering was employed to evaluate the impact of these sonication energies on the particle size distribution.

**Figure 1:**
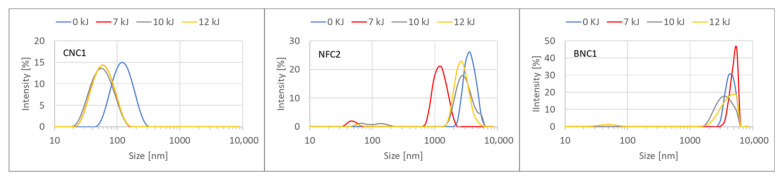
DLS analysis—Impact of sonication energy on particle size distribution for materials CNC1, NFC2, and BNC2.

As depicted in Figure 1, sonication of the crystalline nanocellulose type (CNC1) resulted in a shift in the particle size distribution, with particles presumably deagglomerating. However, no correlation was observed between the level of energy applied to the sample during sonication (7 kJ, 10 kJ, and 12 kJ) and the particle size distribution. This suggests that sonication energies from 7 kJ up to 12 kJ (at 12.5 W) were equally effective for this type of nanocellulose in de-agglomerating the samples without damaging the cellulose structure. For the bacterial and nanofibrillated nanocellulose types, no consistent pattern was observed correlating the particle size distribution to the applied sonication energy. These differences between the three types of nanocellulose are likely due to their structures and varying levels of homogeneity (Section 3.1.4). Since none of the three nanocellulose categories benefited from higher energy levels, it was determined that all samples would be sonicated at 7 kJ.

#### 3.1.2. Dynamic Light Scattering (DLS—Batch Mode)

Dynamic light scattering instruments measure the hydrodynamic radius (or diameter) of particles, which is defined as the radius of a hypothetical hard sphere that diffuses at the same speed as the particles being examined [18]. It is important to note that nanocellulose particles do not have a spherical shape. Therefore, for these materials, the size determined by DLS is only suitable for comparative purposes (e.g., to assess the effect of sample preparation on the particle size distribution or for stability testing), but not for determining the absolute size of the particles. With DLS analysis, various types of information can be obtained. The z-average represents the intensity-weighted mean hydrodynamic size of the ensemble collection of particles measured by DLS. It is derived from a cumulants analysis of the measured correlation curve, assuming a single particle size and applying a single exponential fit to the autocorrelation function. The polydispersity Index (PDI) is extracted as well from the cumulants analysis of the correlation curve and is an indicator of the heterogeneity of particle sizes in the sample. If particles in the population are uniform, the resulting size distribution will be narrow and the PDI small. For heterogeneous particle populations, the size distribution will be broader, and the PDI increases, indicating that the sample is polydisperse. Additionally, by using distribution algorithms in the same measurement, it is also possible to extract size distribution data from DLS data [19].

**Figure 2:**
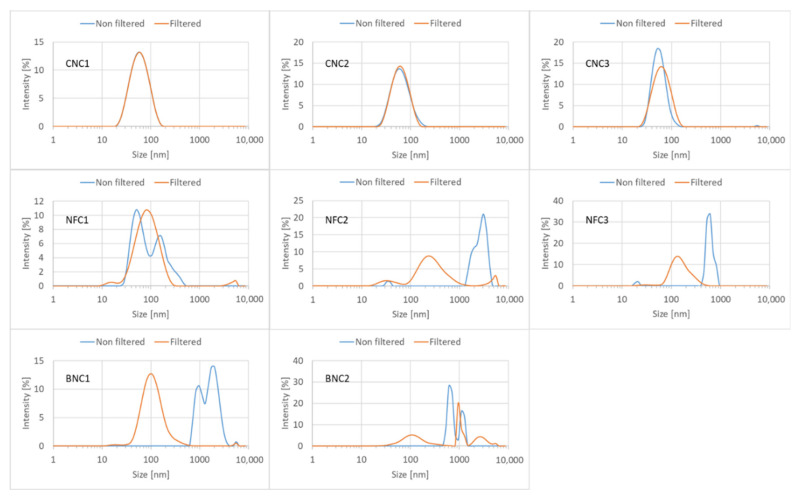
Intensity-based particle size distributions (hydrodynamic diameter of equivalent spheres) for all eight nanocellulose test materials, measured after sonication with 7 kJ, before and after filtration. Data represent the average of three repeat measurements.

Figure 2 presents the hydrodynamic diameter of all eight tested nanocellulose materials before and after filtration. The three crystalline nanocellulose materials (CNC1 to CNC3) have z-averages ranging from 53 to 56 nm and PDIs ranging from 0.145 to 0.155 both before and after filtration, indicating a narrow to moderately polydisperse size distribution [20,21]. Before filtration, the sample obtained by TEMPO oxidation (NFC1) displays a second population at around 200–300 nm. After filtration, this second population is no longer visible, and the remaining particles have a z-average of 73 nm and a PDI of 0.252, which is broader than for the CNC materials but still considered moderately polydisperse. With these four test materials, clear and transparent suspensions were obtained after sonication and filtration, while the other materials remained cloudy with visible agglomerates floating in the suspensions.

Indeed, the fibrillated (NFC) and bacterial nanocellulose (BNC) types, in most cases, exhibited multiple populations and broad size distributions (before filtration), including hydrodynamic diameters exceeding 1 µm. For many of the tested materials (BNC1, NFC2, and NFC3), the filtration step eliminated the fractions containing very large particles. This was confirmed by the gravimetrically determined recovery rates (before vs. after filtration), which were 98.6 ± 1.2% for CNC1, 94.1 ± 9.4% for NFC1, 6.5 ± 0.5% for NFC2, and 1.3 ± 1.2% for BNC1. This indicates that homogeneous materials with shorter fibre lengths achieved nearly complete recovery (~100%), whereas bacterial and fibrillated nanocellulose types—marked by their high aspect ratios and three-dimensional structures—exhibited significantly reduced recovery rates. Nevertheless, the z-averages of the filtered suspensions still ranged from 133 to 4592 nm, and the PDIs ranged from 0.276 to 0.543. Especially, values found for BNC2 exceeded the filter pore size of 450 nm, indicating that agglomeration may have occurred after filtration. The measuring range of DLS typically falls between 1 nm and 10 µm [22], meaning that the presence of even larger particles may go undetected in the unfiltered suspensions. It is important to note that z-average hydrodynamic diameters obtained via cumulants analysis in dynamic light scattering are not reliable for materials with broad size distributions (PDI > 0.3), such as fibrillated and bacterial nanocellulose. The cumulants method assumes a narrow, monomodal distribution and may yield misleading results for polydisperse systems.

#### 3.1.3. Asymmetric Flow Field Flow Fractionation Coupled to MALS and DLS

Following the DLS analysis of all eight test materials, it was decided that AF4 analysis would only be conducted on the nanocrystalline materials (CNC1, CNC2, and CNC3) and the TEMPO-oxidised nanofibrils (NFC1). The other materials exhibited large agglomerates, potentially resulting in steric elution [23] and posing challenges in interpreting the obtained results. Recovery of the injected material is one of the performance criteria outlined in ISO/TS 21362 [12]. The analyte recovery for the four tested materials was 102 ± 10.5%, 90.4 ± 3.4%, 86.4 ± 3.9%, and 84.9 ± 5.4% for materials CNC1, CNC2, CNC3, and NFC1, respectively, in line with the minimum recovery rate of 70% recommended in ISO 21362.

**Figure 3:**
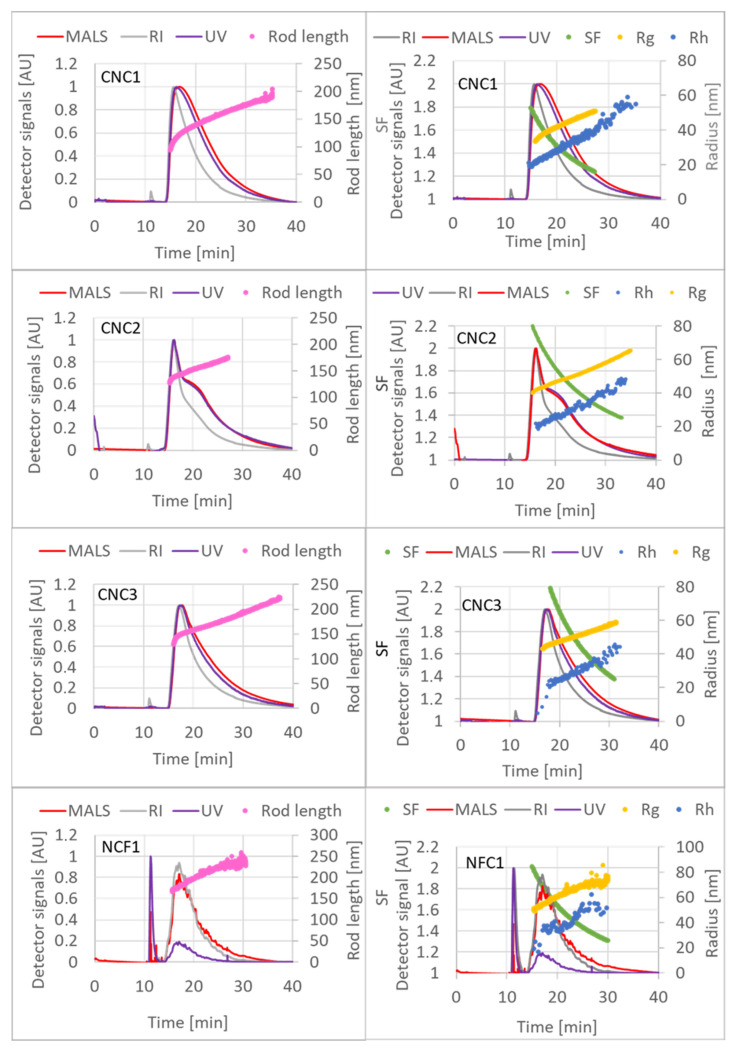
AF4 fractograms of materials CNC1, CNC2, CNC3, and NFC1, (*n* = 2). Left side: The rod length (pink line) is overlaid over the 90° MALS detector signal (red continuous solid line), the RI signal (grey continuous solid line), and the UV signal (purple continuous solid line). Right side: The hydrodynamic radius (Rh, blue dots) and the radius of gyration (Rg, yellow dots) are overlaid over the 90° MALS detector signal (red continuous solid line), the RI signal (grey continuous solid line), and the UV signal (purple continuous solid line). The shape factor is plotted as green line.

Figure 3 displays the fractograms and associated sizing values obtained for the four injected materials. Materials CNC1, CNC2, and CNC3 exhibit comparable elution profiles, with hydrodynamic radii (Rh) ranging from around 20 to 55 nm and radii of gyration (Rg) ranging from approximately 35 to 65. In contrast, the fractogram for material NFC1 generally shows noisier signals for all detectors, a distinct void-peak, and slightly higher size values for Rh and Rg, consistent with the DLS results (Section 3.1.2). Particle rod-lengths can be extracted from light-scattering data by applying a dedicated model. This rod model assumes that the thickness of a rod-shaped particle is negligible compared to its length. If known, the software allows it; however, for the input of rod width, it provides a more accurate analysis. In this case, a thickness of 10 nm, as determined by TEM (Section 3.1.4), was used in the software. The resulting rod lengths for materials CNC1, CNC2, and CNC3 ranged from approximately 100 to 200 nm, which is in good agreement with the Feret lengths determined by TEM (Section 3.1.4) and the findings of Mukherjee and Hackley [11] (101–204 nm), although the materials analysed were not exactly the same. Notably, material NFC1 exhibited the highest rod length, spanning 150–225 nm.

While neither the hydrodynamic radius (or diameter) nor the radius of gyration provides direct information on the size of rod-like particles [24], the ratio of these two measurements provides insight into the shape factor (SF = Rg/Rh) [25]. A ratio of 0.77 is associated with monodisperse compact spheres; a ratio of around 1 is compatible with hollow spheres with a thin shell, a ratio ranging from 1.3 to 1.6 defines coil structures and rod-like shapes, and a ratio between 1.8 and 2.25 defines prolate ellipsoid shapes [26]. Figure 3 (right side) shows how shape factors for all four materials range from approximately 1.3 to 2, indicating a rod-like and/or prolate ellipsoid shape of the particles, confirmed also by TEM analysis (Section 3.1.4). Comparable values were found in a study conducted by Mukherjee and Hackley [11], in which a material corresponding to sample CNC2 was used.

#### 3.1.4. Transmission Electron Microscopy (TEM)

**Figure 4:**
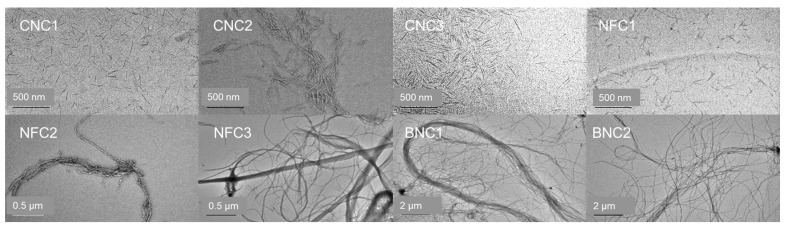
Representative TEM images of the eight tested nanocellulose types.

**Figure 5:**
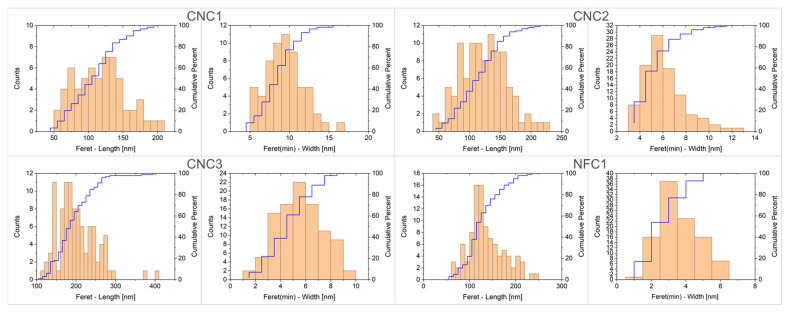
Size distribution histograms for fibre-length and fibre-width determined with TEM for materials CNC1, CNC2, CNC3, and NFC1. The blue line represents the cumulative percentage of the size distribution.

All eight nanocellulose test materials were analysed using transmission electron microscopy, one of the few analytical techniques capable of providing two-dimensional sizing data of rod-shaped particles. The TEM images presented in Figure 4 clearly illustrate why the crystalline nanocellulose (CNC1 to CNC3) types and the TEMPO-oxidised nanofibrils (NFC1) exhibited a more homogeneous particle size distribution when measured with DLS and AF4 compared to the other four materials. The nanocrystals in materials CNC1, CNC2, and CNC3, as well as the particles obtained by the TEMPO-treated nanofibrils, all have a particle length ranging from 50 to 300 nm and a particle width ranging from 2 to 15 nm (Figure 5), resulting in an average aspect ratio (length/width) of 10.

This aligns well with the findings of previous studies [14,15,27]. In contrast, the non-TEMPO-treated fibrillated (NFC2 and NFC3) and bacterial nanocellulose (BNC1 and BNC2) types exhibit a broad range of fibre lengths, often exceeding 1 µm.

Measuring the fibre width was challenging due to the clustering of various fibres, making it difficult to measure them individually. Determining the particle size distribution for length and width was not feasible for these materials. The morphology of these materials explains the difficulty in analysing them with AF4 and DLS.

A table summarising all sizing values for the nanocellulose test samples is provided in the Supplementary Materials (SM3).

### 3.2. Bacterial Nanocellulose Extracted from Food Products

Following the characterisation of various types of nanocellulose test materials described in the preceding sections, the subsequent sections outline the outcomes of the assessment to determine whether it is possible to identify bacterial nanocellulose in real food products sourced from the market. To this end, a multi-tiered approach was employed, utilising pyGC-MS for the identification of cellulose, transmission electron microscopy (TEM) to confirm that the material’s width falls within the nano-size range, and X-ray diffraction (XRD) to ascertain that the nanocellulose is of bacterial origin.

#### 3.2.1. Pyrolysis GC-MS of Cellulose Extracted from Nata de Coco Containing Food Samples

The primary compounds generated during the pyrolysis of cellulose under the conditions described in Section 2.2.2 are 1,6-anhydro-β-D-glucopyranose (levoglucosan, LG), 1,6-anhydro-α-D-galactofuranose (HGF), and 1,4:3,6-Dianhydro-α-D-glucopyranose (DHGP) [28,29], with levoglucosan being by far the most abundant compound. It retains much of the molecular structure of its monomer precursor, glucose, and is therefore considered a pyrolytic marker for glucose units [28]. Among other ingredients, the investigated samples contained sugar (sucrose). During pyrolysis under the given conditions, reducing sugars such as sucrose, glucose, and fructose are known to form levoglucosan, which can complicate the unequivocal identification of (nano)cellulose in a sample when they are present together.

**Figure 6:**
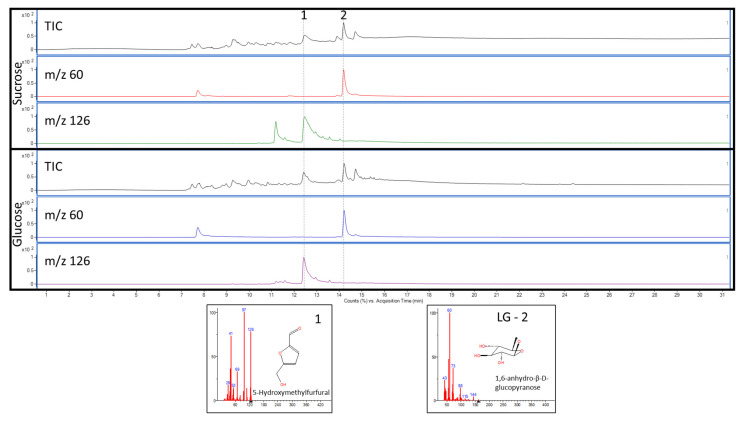
Pyrograms of sucrose and glucose. The total ion current chromatogram, as well as extracted ion chromatograms for *m*/*z* 60 (levoglucosan) and *m*/*z* 126 (5-Hydroxymethylfurfural), are shown.

**Figure 7:**
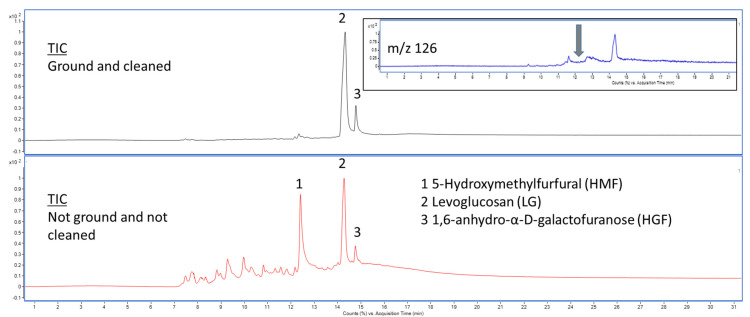
Pyrograms of ground/cleaned (**upper**) and unground/uncleaned (**lower**) nata de coco from the pudding product. Both pyrograms are presented as total ion current chromatograms. The insert in the upper pyrogram shows the extracted ion chromatograms for *m*/*z* 126, the characteristic fragment for HMF. The grey arrow indicated the retention time at which HMF elutes.

However, reducing sugars also produces 5-(hydroxymethyl)furfural (HMF) [30], which can therefore be employed as a proxy to detect the presence of sucrose. Figure 6 illustrates the pyrograms of sucrose and glucose, showing that reducing both sugars yields levoglucosan (extracted ion *m*/*z* 60) and HMF (extracted ion *m*/*z* 126). In contrast, cellulose does not generate HMF, as it is unable to readily form acyclic structures due to the presence of only a single reducing sugar at one end of the polymer chain. Instead, cellulose predominantly cyclises to anhydrosugars, such as levoglucosan and anhydro-oligosaccharides [31]. To evaluate the effectiveness of the sucrose removal during the clean-up procedure described in Section 2.2.1, the presence of HMF was monitored. Figure 7 presents the pyrograms for both the ground and cleaned nata de coco cube and the unground/uncleaned cube contained in the pudding product. The cleaning procedure is shown to be highly efficient, as evidenced by the overall lower abundance of pyrolysis products and, more notably, the absence of HMF in the pyrogram.

Following the removal of sugar, the presence of levoglucosan and 1,6-anhydro-α-D-galactofuranose (HGF) can therefore be used to unequivocally identify cellulose in a sample. The pyrograms of cleaned nata de coco from Mogu Mogu, SCOBY, and the kombucha tea residue resembled those depicted in Figure 7 and are provided in the Supplementary Materials (SM4). Different from all other pyrograms, an additional dominant peak appeared exclusively in the kombucha tea residue’s pyrogram. The associated compound was identified as D-allose (CAS# 2595-97-3). One possible explanation is its formation as a by-product of the Maillard or caramelisation reaction. D-allose may arise as a transient intermediate or side product during polyphenol–sugar condensation reactions [32]. The presence of polyphenols in kombucha [33] creates a biochemically complex matrix that could influence carbohydrate thermal degradation pathways, including those producing rare sugar epimers such as D-allose. 

The use of pyrolysis GC–MS proved to be a suitable analytical technique for cellulose identification. However, the profile of compounds produced during pyrolysis does not provide sufficient information to distinguish between crystalline nanocellulose (CNC), bacterial nanocellulose (BNC), and nanofibrillated cellulose (NFC).

#### 3.2.2. Transmission Electron Microscopy of Cellulose Extracted from Nata de Coco-Containing Food Samples, from SCOBY and from the Kombucha Tea Residue

**Figure 8:**
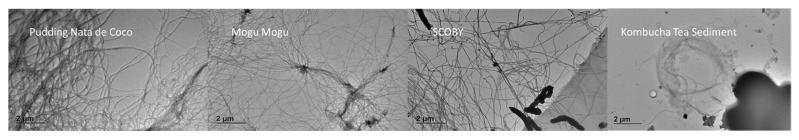
Representative TEM images of nata de coco from pudding, Mogu Mogu, SCOBY, and the sediment of kombucha tea.

Figure 8 presents transmission electron microscopy (TEM) images of purified nata de coco samples derived from pudding and Mogu Mogu, alongside those of the SCOBY test material and the sediment from kombucha tea. All four samples exhibit filaments with widths that fall within the nanoscale range. A qualitative comparison of the filament-width size distributions reveals that nata de coco extracted from the pudding product exhibits a slightly broader range compared to nata da coco from the Mogu Mogu drink. This variation in filament width may account for the need for distinct cleaning procedures for nata de coco obtained from the pudding product versus the Mogu Mogu drink.

In the TEM image of the SCOBY product, bacteria are visible, which is to be expected as they form part of this product, comprising a starter culture of bacteria and yeast used to produce the kombucha drink. Notably, filaments in the nano size range were also detected in the kombucha tea sediment, suggesting that the sediment likely retains residual remnants of the SCOBY starter culture, as indicated by the ingredients listed on the label (Table 3). As described in Section 3.1.4, both fibrillated and bacterial nanocellulose exhibit a wide range of fibre lengths, making it challenging to distinguish between these two types using TEM. An alternative analytical technique that enables differentiation between fibrillated and bacterial cellulose is X-ray diffraction (XRD), which can be used to determine the crystallinity index (CI) [34].

#### 3.2.3. Determination of Crystallinity Index of Food Samples with X-Ray Diffraction (XRD)

As part of the NanoCellUp project [9], the crystallinity of the same eight nanocellulose test materials used in this study was determined, revealing a clear trend between the samples according to the synthesis methodology (crystalline vs. nanofibrillated vs. bacterial). The bacterial nanocellulose materials exhibited the highest crystallinity (around 93%), followed by the crystalline nanocellulose materials (84–87%), while the nanofibrillated materials had the lowest crystallinity (43–52%) [35].

**Figure 9:**
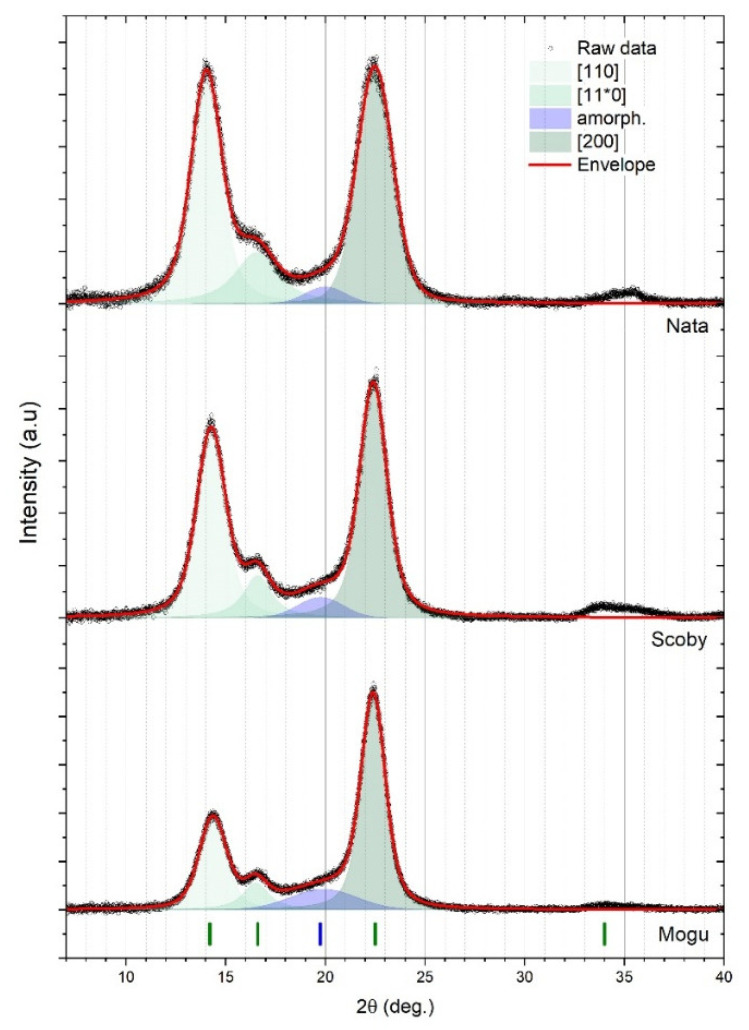
XRD spectra and spectral deconvolution for the crystalline and amorphous components for the three tested nanocellulose food materials (from **top** to **bottom**: nata de coco from pudding; SCOBY; nata de coco from Mogu Mogu). The green vertical lines at the bottom indicate the peak centre positions for cellulose type Ib structure from the reference [36]. The blue vertical line indicates the position of the amorphous band.

In this study, the diffractograms of the three tested food materials showed the characteristic cellulose reflection sets, but the relative intensities of different reflections and the total scattered intensity varied across the samples. Deconvoluted individual peaks (green and blue Lorentzian components in Figure 9) centres match reference assignments for the specific “cellulose I” polymorph. Assignment of the arrangement of the hydrogen bond network type (i.e., cellulose Iα and Iβ polymorphs) can be made within the limits of the precision in the acquired data. The most likely assignment, based on the observed peaks’ intensity ratios, is a mix of phases with Iβ present as the dominant component. Main Miller indices’ reflections of monoclinic Iβ structure are (110), (11*0), (200), and (004), and their calculated locations [35] match well with the experimentally observed reflections located at 14.8°, 16.6°, 22.6°, and 34.1°, respectively. After background and air scattering signal subtraction from all diffractograms, a contribution from the broadband reflection peak in the 16–24° diffraction region result is still evident in the raw data. This residual intensity is likely originating from amorphous material within a mostly crystalline sample. In all investigated cases, it was not possible to fully reproduce measured data using only the combination of the three cellulose Iβ Lorentzian line-shapes (i.e., by using an idealized fully crystalline nanocellulose spectrum). Consistent matches of the peak fit envelope with measured data were possible only by adding (at least) an additional amorphous peak, positioned in the interval 18–22°. For consistency, the position was fixed at 20° in all analyses. The integral intensity of the amorphous contribution to the signal varies from sample to sample. Being more intense in the nata de coco from the Mogu Mogu drink and less intense in the nata de coco from the pudding. The diffractograms’ Scherrer’s analysis also shows a larger (200) crystallite size of 6.6 ± 0.2 nm for the nata de coco Mogu Mogu samples, while the crystalline domains are smaller in the SCOBY (c_s_= 5.9 ± 0.2 nm) and the nata de coco from the pudding sample (c_s_= 4.3 ± 0.2 nm). Also, the intensity ratio between different crystal planes differs across samples and from the idealized cellulose Ib structure, indicating a certain degree of anisotropic crystallization in the investigated materials. The crystallinity index of the three tested food materials determined in this study was measured according to the same protocol used in [34] and resulted to be, on average, 95.7 ± 0.9%, 84.5 ± 3.6%, 81.1 ± 0.8% for nata de coco from pudding, SCOBY starter culture, and nata de coco from the Mogu Mogu drink, respectively. Representative diffractograms, including the peak deconvolution used to determine the crystallinity index for the three tested samples, are included in Figure 9. The obtained values are consistent with those reported in other studies, which range from 82% to 91% [34,36,37].

**Figure 10:**
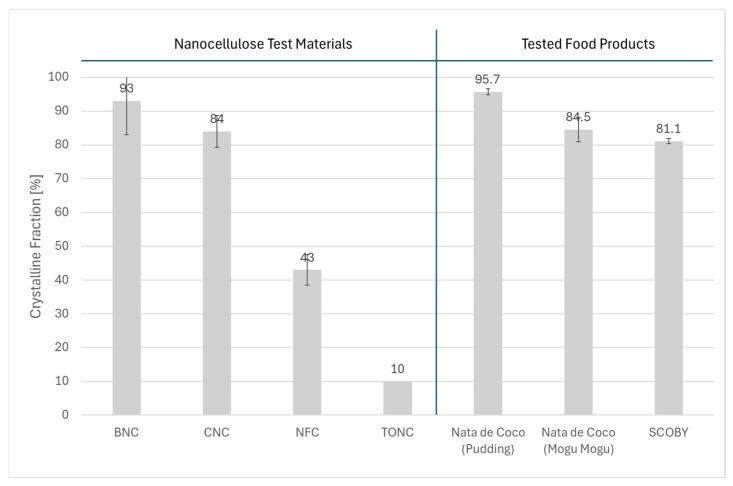
Comparison of crystallinity indices between reference nanocellulose materials and tested food products.

Notably, the crystallinity index of bacterial and crystalline nanocellulose is relatively similar, whereas that of fibrillated nanocellulose is significantly different (Figure 10). TEM images enabled us to rule out the presence of crystalline nanocellulose in the food test materials. By process of elimination, we could confidently identify the presence of bacterial nanocellulose in the three food materials.

**Figure 11:**
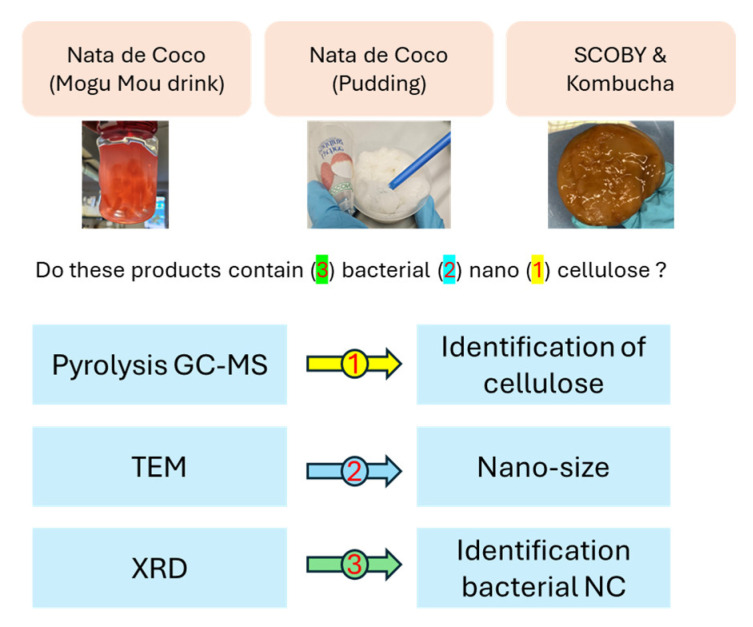
Multi-tiered approach for the identification of bacterial nanocellulose in food-related products.

In conclusion, the combination of pyrolysis GC-MS, TEM, and XRD enabled the unequivocal identification of bacterial nanocellulose in both nata de coco materials and the SCOBY starter culture. This multi-tiered analytical approach is depicted in Figure 11.

## 4. Conclusions

The study encompasses two distinct yet interconnected activities: (1) the characterisation of eight nanocellulose test materials, including crystalline, fibrillated, and bacterial nanocellulose, using various analytical techniques. While characterisation itself does not directly constitute a risk assessment, the data it generates are an essential part for assessing risks—not only in food products, but also in other applications. The results demonstrate that different types of nanocellulose can be identified/distinguished by their unique physicochemical properties using a combination of analytical techniques. In the second activity (2), a case study investigated bacterial nanocellulose in commercially available food-related products, including pudding and beverages containing nata de coco, SCOBY, and kombucha. A multi-method approach was employed, integrating pyrolysis GC-MS, transmission electron microscopy (TEM), and X-ray diffraction (XRD), leveraging the differences in properties outlined in the first part of the study. While pyrolysis GC-MS provided general cellulose identification, TEM revealed particle morphology (size, shape, and aspect ratio), and XRD analysed crystallinity—key parameters for distinguishing bacterial, crystalline, and fibrillated cellulose.

This approach may prove relevant should food products containing bacterial nanocellulose enter the European market as novel foods in the future. Should the European Commission authorise such nanocellulose-based novel foods, an implementing act would be published. This act would outline the specifications and uses under which the novel food is authorised, against which European Member State control laboratories would conduct compliance testing. The tiered methodology presented here could effectively inform market surveillance methodologies, potentially impacting the obligations of European Member State control laboratories. One limitation of the presented approach is the availability and practicality of the proposed analytical techniques: some analytical techniques employed in this approach are advanced and often lack routine availability in regulatory or industrial laboratories. However, identifying bacterial nanocellulose requires simultaneous assessment of chemical identity, morphology (shape and size), and crystallinity. Therefore, a primary challenge for the future is identifying more widely accessible techniques that comprehensively address all these critical parameters. Notably, in 2018, the European Commission received a request to authorise the placement of a bacterial cellulose aqueous suspension on the EU market as a novel food ingredient. This request was withdrawn in early summer 2025. While this demonstrates that applications involving nanocellulose in the food sector are currently under development, it also underscores that such innovations are likely to reach the market within the foreseeable future.

## Figures and Tables

**Table 1:** Nanocellulose test materials used in this study, their providers, and the content of cellulose.

Material ID	Test Sample Material Identification Code	Type of Nanocellulose	Provider, Product Code, and Manufacturing Process (Where Available)	Concentration of Nanocellulose [%, *w*/*w*]
1	CNC1	Cellulose nanocrystals	University of Maine Process Developer Center (PDC) Derived from wood pulp (eucalyptus) using sulfuric acid.	10.6% (106 mg mL^−1^)
2	CNC2	Cellulose nanocrystals	CelluForce Product Code: NCV100-NAL90 Derived from wood pulp using sulfuric acid.	6% (60 mg mL^−1^)
6	CNC3	Cellulose nanocrystals	Nanografi Product Code: NG01NC0102 Derived from pine wood using sulfuric acid.	6% (60 mg mL^−1^)
4	NFC1	TEMPO-oxidized FPL ^1^ Cellulose Nanofibrils	University of Maine Process Developer Center (PDC) TOCN Carboxylation of pulp nanofibril surface using the catalyst TEMPO.	1% (10 mg mL^−1^)
5	NFC2	Cellulose nanofibers	University of Maine Process Developer Center (PDC) St.Felician pulp from Domtar’s mill in Canada. This facility produces market pulp used in various paper products. Mechanical refiners are then used to process further.	3% (30 mg mL^−1^)
7	NFC3	Cellulose nanofibers	Sappi Valida S231C Derived from wood pulp. The cellulose fibres are mechanically processed to create a highly fibrillated structure.	3% 30 mg mL^−1^
3	BNC1	Bacterial nanocellulose	Material donated by a Portuguese research centre.	1.04% (10.4 mg mL^−1^)
8	BNC2	Bacterial nanocellulose	Cass Materials Pty Ltd., Perth, Australia Bacterial nanocellulose dry sheets (Nata de coco) from Dr. Gary Cass (founder/CEO of Cass Materials Pty Ltd., Perth, Australia), Product Code: BNC-01-AU.	0.5% (5 mg mL^−1^)

^1^ Derived from filter paper linter pulp.

**Table 2:** Settings in DLS software (Zetasizer Version 8.02).

Parameter	Setting
Dispersant	Aqueous sodium chloride solution (5 mM)
Dispersant refractive index	1.330 @ 20 °C The correct refractive index for the selected measurement temperature was automatically set/adjusted by the software
Evaluation algorithm for distribution analysis	General Purpose
Measurement temperature	The instrument is capable of thermal regulation. The temperature in the laboratory was measured before each measurement and set accordingly in the software. This allowed optimising the equilibration time. Set temperatures ranged from 20 to 23 °C.
Temperature equilibration time	180 s
Refractive index	Not set, since interested only in intensity-based size distribution
Absorption	

**Table 3:** Ingredients of tested food products.

Sample ID	Product	Declared Ingredients
A	Mogu Mogu Drink	Water, coconut jelly, sugar, fructose, E330 (citric acid), E327 (calcium lactate), aroma, E211 (sodium benzoate), E418 (gellan gum), E129 (Allura red AC)
B	Pudding Nata de Coco	Water, nata de coco, sugar, fruit juice, skimmed milk powder, seaweed extract, citric acid, aroma, colorants E129 and E102
C	Kombucha SCOBY Starter Kit	SCOBY (bacteria and yeast), tea (residues), sugar
D	Kombucha Green Tea	Karma Kombucha (filtered water, blond cane sugar, green tea, kombucha culture), selected live culture, and carbon dioxide resulting from fermentation. Contains traces of alcohol (<1.2%).

## Data Availability

The raw data supporting the conclusions of this article will be made available by the authors on request.

## Supplementary Materials

The following supporting information can be downloaded at: https://www.mdpi.com/article/10.3390/nano15201565/s1, SM1: Determination of ‘Delivered Acoustic Power’ of the probe sonicator used; SM2: Food products containing bacterial nanocellulose; SM3: Sizes determined with DLS (batch mode), AF4-DLS/MALS, and TEM for the eight nanocellulose test materials; SM4: Pyrograms of nata de coco from Mogu Mogu, SCOBY and Kombucha tea sediment.
