# Supplementary material for: Characterisation of Nanocellulose Types Using Complementary Techniques and Its Application to Detecting Bacterial Nanocellulose in Food Products
Source: Nanomaterials (Basel). 2025 Oct 14;15(20):1565. doi: 10.3390/nano15201565 (PMC12566323; doi:10.3390/nano15201565)
Supplement: Supplementary file 1 [file nanomaterials-15-01565-s001.zip › SM4 Pyrograms Mogu Mogu SCOBY KOMBUCHA_150625.docx]

**Supplementary material – SM4**

**Pyrograms of nata de coco from Mogu Mogu, SCOBY and Kombucha tea sediment**

The pyrograms of nata de coco from the Mogu-Mogu drink and from SCOBY, shown below (figure 1), demonstrate that the vast majority of sugar has been successfully removed by the cleaning procedure.


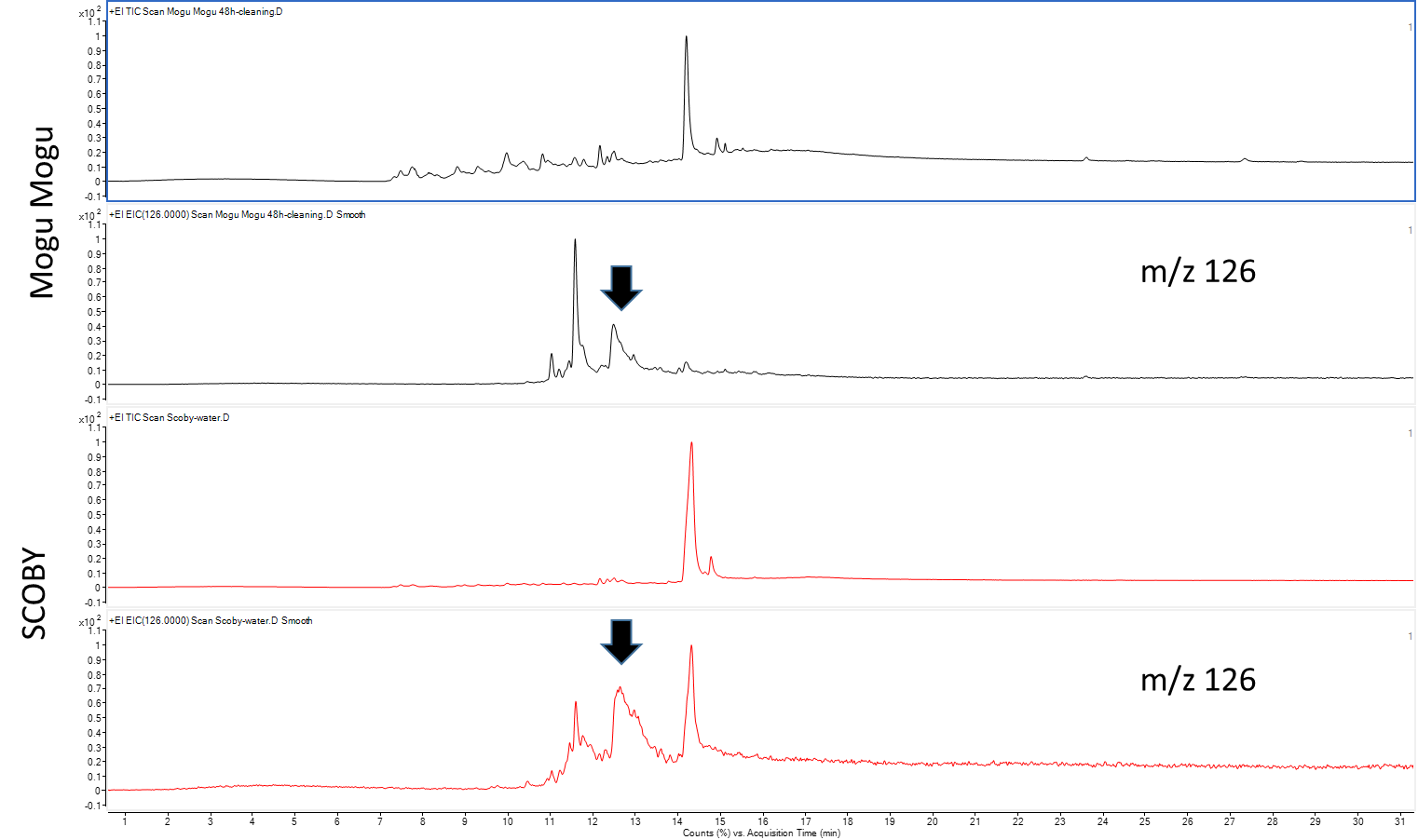


**Figure 1.** Pyrograms of nata de coco from Mogu Mogu drink and from SCOBY. The respective upper pyrograms are in total ion current (TIC) and the respective lower pyrograms in selected ion mode (m/z 126) for the detection of 5-HMF

As detailed in the manuscript, the pyrogram of the Kombucha tea sediment exhibits a distinct peak that is absent in the pyrograms of the other tested samples. This peak has been identified as D-allose.


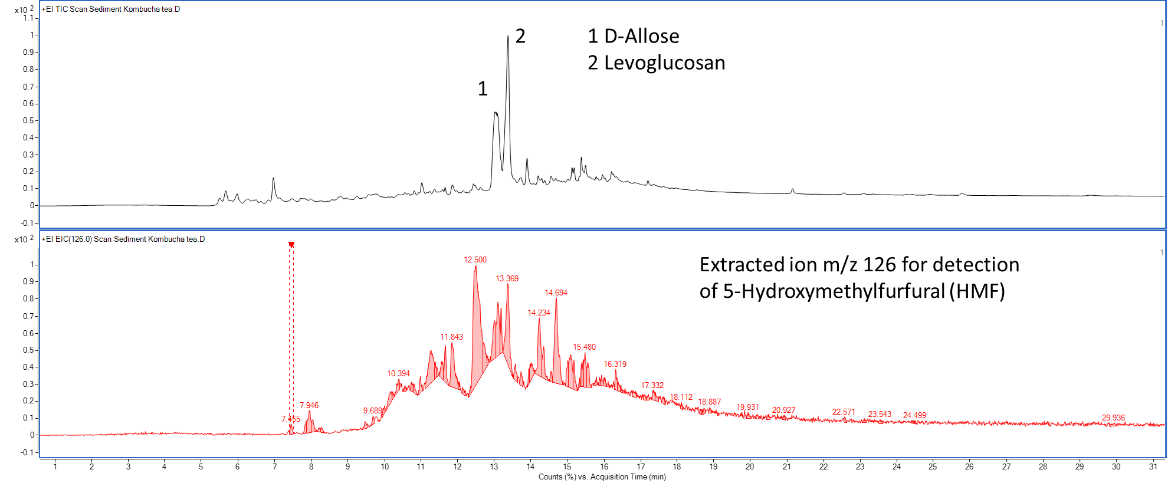


**Figure 2.** Pyrograms of kombucha tea sediment. Upper pyrogram in total ion current (TIC); lower pyrogram in selected ion mode (m/z 126) for the detection of 5-HMF

Please note that the retention times between the Mogu Mogu and SCOBY samples and the kombucha tea sediment sample have shifted as a result of a required column change.
