# Supplementary material for: Characterisation of Nanocellulose Types Using Complementary Techniques and Its Application to Detecting Bacterial Nanocellulose in Food Products
Source: Nanomaterials (Basel). 2025 Oct 14;15(20):1565. doi: 10.3390/nano15201565 (PMC12566323; doi:10.3390/nano15201565)
Supplement: Supplementary file 1 [file nanomaterials-15-01565-s001.zip › SM1 Characterisation sonication tip_090725.docx]

**Supplementary Material SM1**

**Determination of the ‘Delivered Acoustic Power’ of the probe sonicator used (3mm tip)**

Procedure:

- 365.6 mL were weighed into a 400 mL beaker
- The sonicator was immersed in the beaker
- A temperature probe was immersed in the beaker
- A magnetic stirring bar was added to the beaker and the magnetic stirrer was tuned on
- Probe sonicator was turned on and the temperature was recorded every 30 seconds over a time-period of 5 minutes


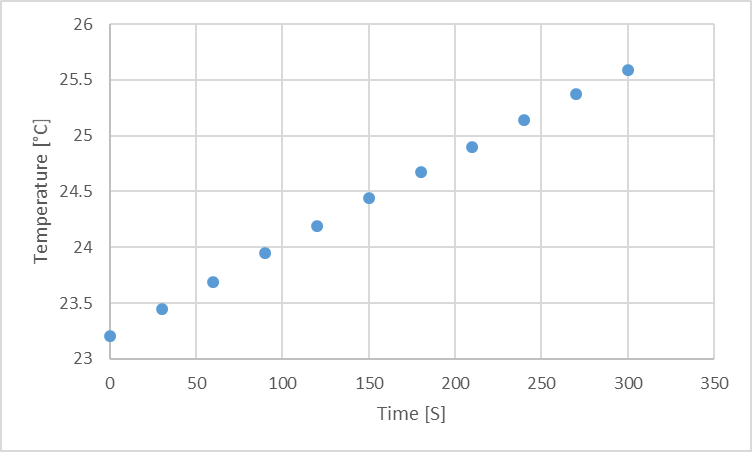
The following values were obtained:

| Time  [s] | Temperature  [°C] |
| --- | --- |
| 0 | 23.2 |
| 30 | 23.45 |
| 60 | 23.69 |
| 90 | 23.95 |
| 120 | 24.19 |
| 150 | 24.44 |
| 180 | 24.67 |
| 210 | 24.9 |
| 240 | 25.14 |
| 270 | 25.37 |
| 300 | 25.59 |

Power = (dT/dt) x M x C
 = (2.39K/300s) x 365.6g x 4.186 (J/gxK)
 = 12.5 Watt [J/s]

P : Delivered acoustic power [W]
T : Increase of temperature [K]
t : Time interval [s]
M : Mass of water [g]
C : Specific heat of water [4.186 J/gxK]
