# Supplementary material for: Characterisation of Nanocellulose Types Using Complementary Techniques and Its Application to Detecting Bacterial Nanocellulose in Food Products
Source: Nanomaterials (Basel). 2025 Oct 14;15(20):1565. doi: 10.3390/nano15201565 (PMC12566323; doi:10.3390/nano15201565)

**Supplementary material – SM2**

**Food products containing bacterial nanocellulose**

The following food products (or in the case of SCOBY, products used to modify food) were purchased and subsequently analysed as part of this study.

**1) Mogu Mogu Drink (Nata de Coco)**


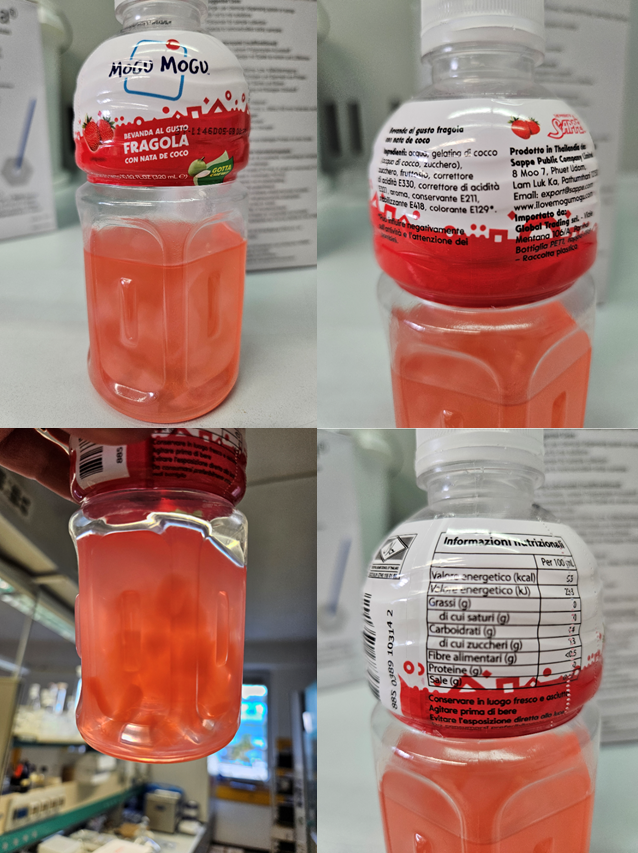


**2) Pudding Nata de Coco (Nata de Coco)**

The pudding contains cubes immersed in a softer cream.
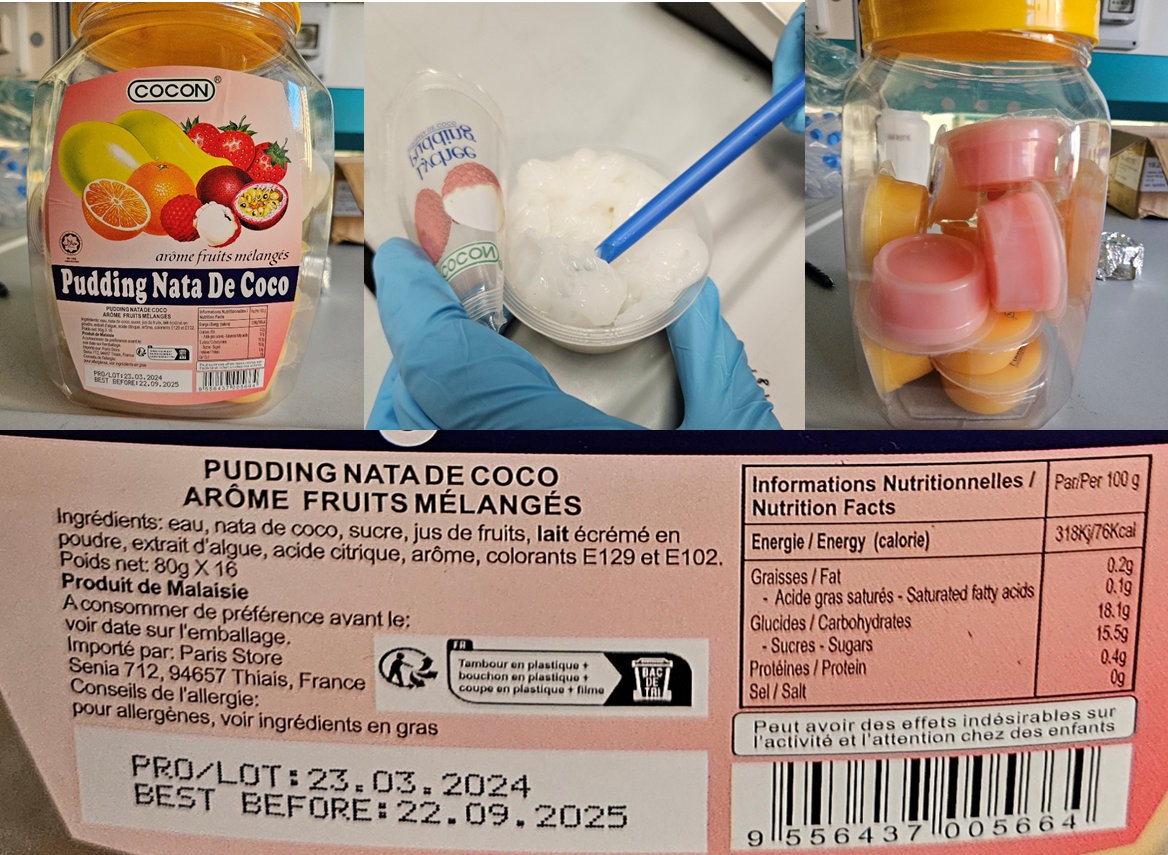


**3) Kombucha SCOBY Starter Kit**


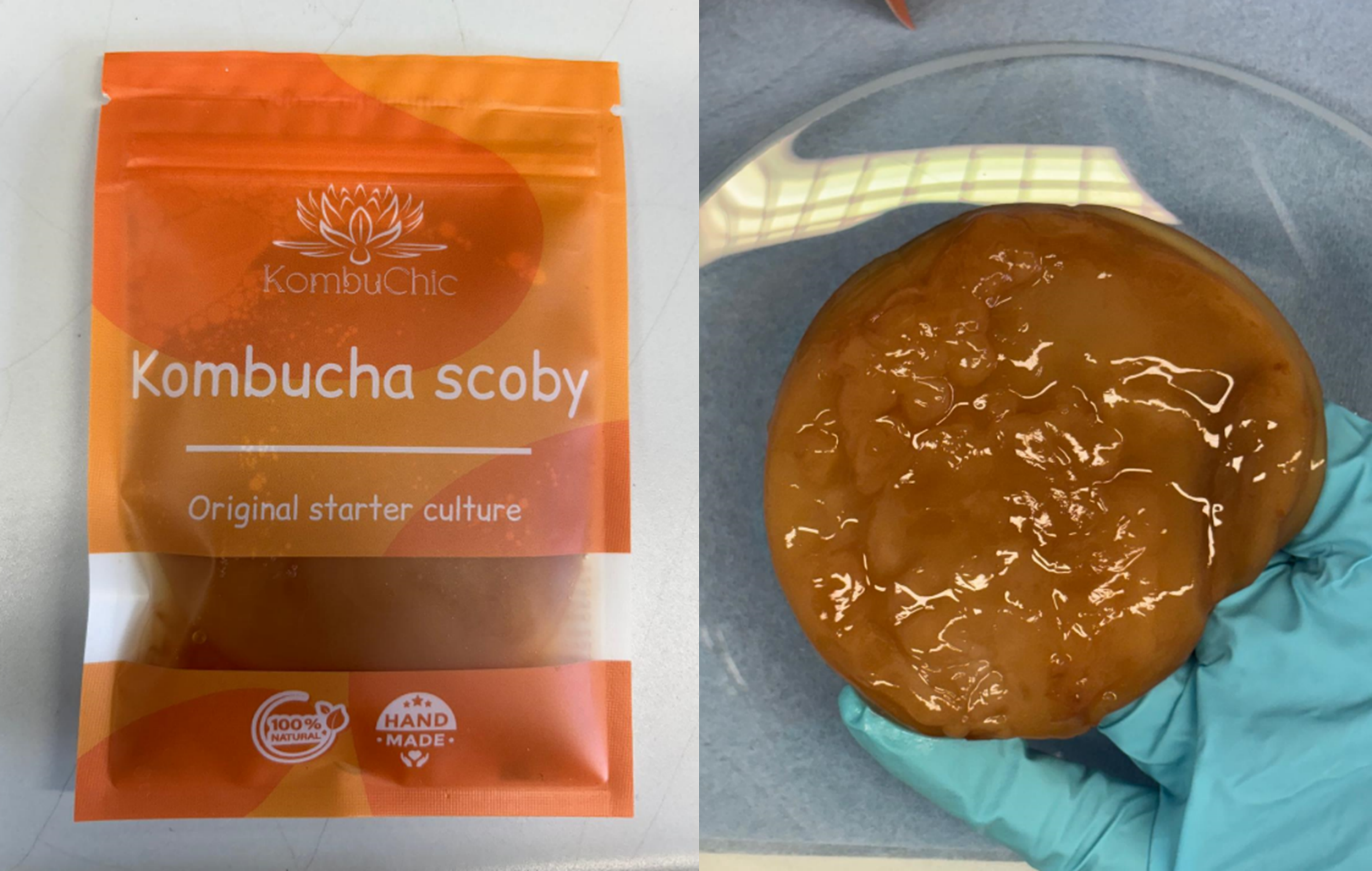


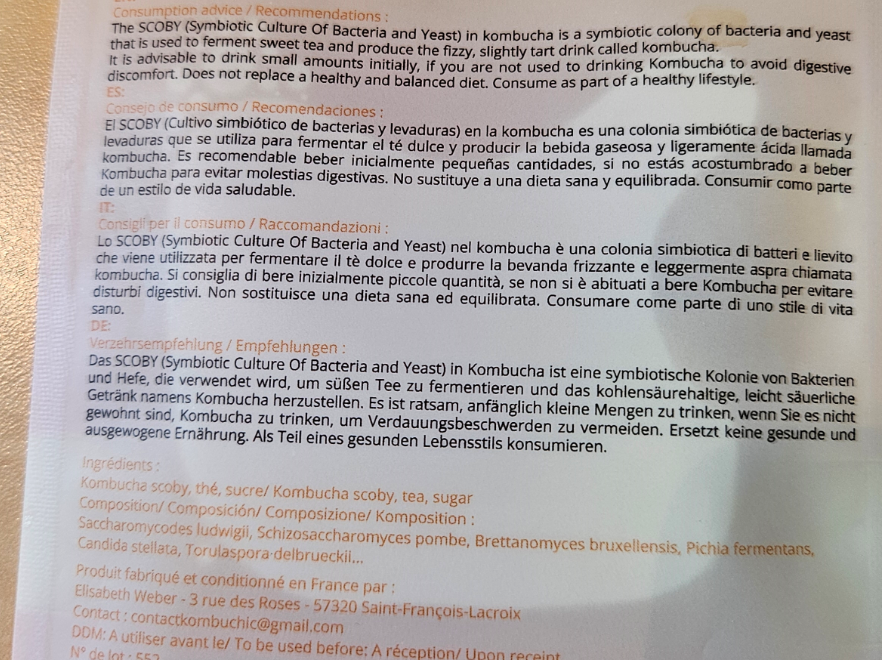


**4) Kombucha Green Tea**


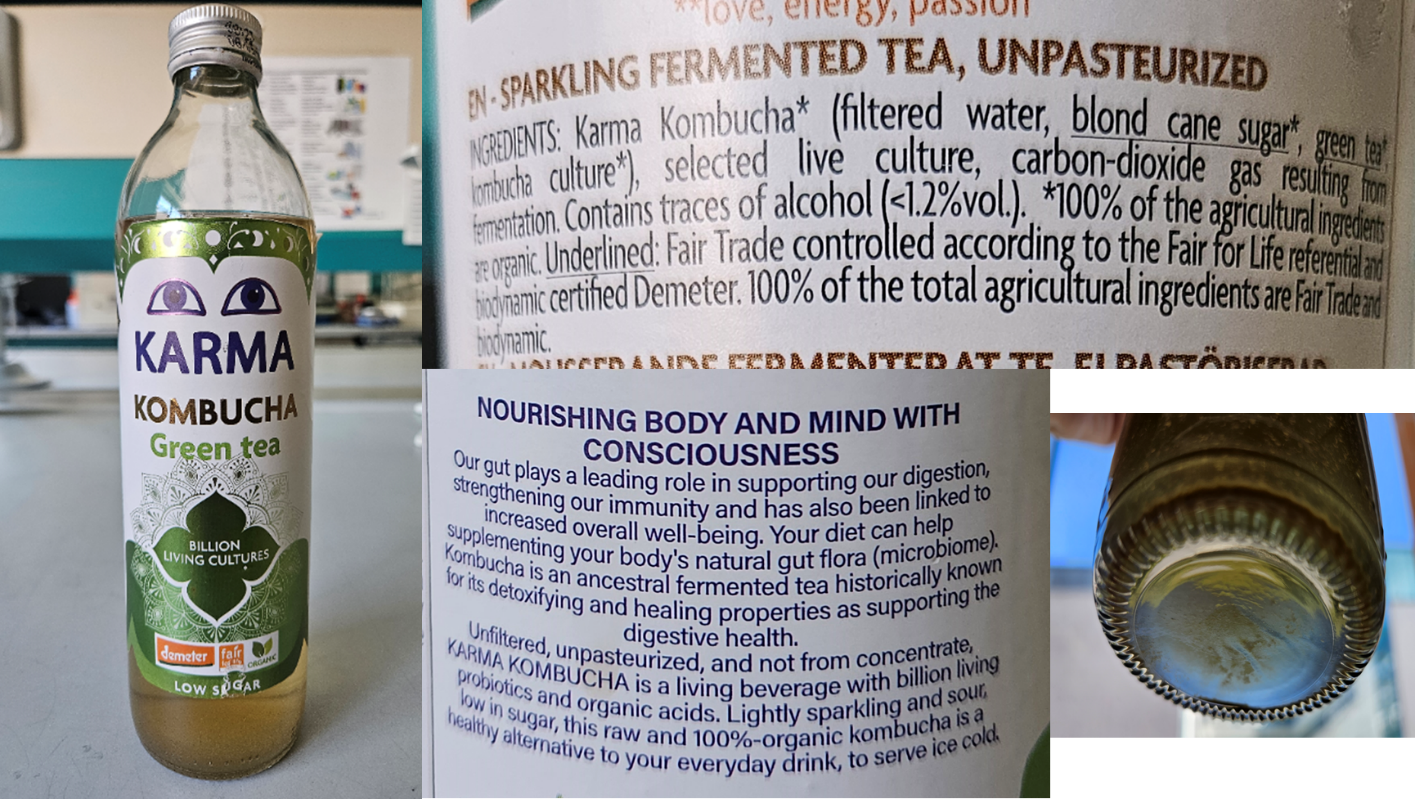

Supplement: Supplementary file 1 [file nanomaterials-15-01565-s001.zip › SM2 Food products pictures_090725.docx]
