# Supplementary material for: Characterisation of Nanocellulose Types Using Complementary Techniques and Its Application to Detecting Bacterial Nanocellulose in Food Products
Source: Nanomaterials (Basel). 2025 Oct 14;15(20):1565. doi: 10.3390/nano15201565 (PMC12566323; doi:10.3390/nano15201565)
Supplement: Supplementary file 1 [file nanomaterials-15-01565-s001.zip › SM3 Sizing comparison_150925.docx]

**Supplementary material – SM3**

**Sizes determined with DLS (batch mode), AF4-DLS/MALS, and TEM for the eight nanocellulose test materials**

| Material Identification Code | DLS batch mode (after filtration)  [Dh, nm] and PDI | AF4-DLS  [Rh, nm] | AF4-MALS [Rg, nm] | AF4-MALS  (Length with Rod model) [nm] | TEM Feret(min) [nm] | TEM Feret [nm] |
| --- | --- | --- | --- | --- | --- | --- |
| CNC 1 | Dh: 53 – 56  PDI: 0.145-0.155  (n=3) | 20-55 (n=3) | 35-65 (n=3) | 100-200 (n=3) | 2-15 | 50-300 |
| CNC 2 |  |  |  |  |  |  |
| CNC 3 |  |  |  |  |  |  |
| NFC 1, TEMPO oxidised | Dh: 73 PDI: 0.252 (n=3) | Slightly higher compared to CNC1-3 | | 150-225 (n=3) | 1-6 | 50-220 |
| NFC 2 | Data not reliable | Not analysed | Not analysed | Not analysed | Not analysed | Frequently exceeds 1 µm |
| NFC 3 |  |  |  |  |  |  |
| BNC 1 |  |  |  |  |  |  |
| BNC 2 |  |  |  |  |  |  |
